# A new species of *Wesmaelius* Krüger from Mexico, with a key to the New World species of the subgenus *Kimminsia* Killington (Neuroptera, Hemerobiidae)

**DOI:** 10.3897/zookeys.841.29570

**Published:** 2019-04-23

**Authors:** Yesenia Marquez-López, Atilano Contreras-Ramos

**Affiliations:** 1 Maestría en Biología, Universidad Autónoma Metropolitana-Iztapalapa, Departamento de Biología, 09340 Ciudad de México, Mexico Universidad Autónoma Metropolitana-Iztapalapa Ciudad de México Mexico; 2 Instituto de Biología-UNAM, Departamento de Zoología, Ciudad Universitaria, 04510 Ciudad de México, Mexico Ciudad Universitaria Ciudad de México Mexico

**Keywords:** Brown lacewings, taxonomy, Tlaxcala, Transmexican Volcanic Belt

## Abstract

Wesmaelius (Kimminsia) nanacamilpa Marquez & Contreras, **sp. n.**, a brown lacewing from Tlaxcala state, Mexico is described and illustrated. This is the second recorded species of *Wesmaelius* from Mexico, and the third from Middle America. Males of the new species may be identified by parameres separate apically, styliform sclerites directed basally, as well as a rounded gonarcus with a short entoprocessus. Females may be distinguished from closely related species by a subgenital plate with the central plate broadly incised basally. There are now 16 species of *Wesmaelius* known from the New World.

## Introduction

*Wesmaelius* Krüger is a mostly Holarctic brown lacewing genus, with highest diversity in temperate regions of Eurasia and North America (Yang 1980, Oswald 1993). It includes 65 valid species worldwide (Zhao et al. 2017, Oswald 2018). Thirteen species are known from the United States and Canada, with one more from the south-western United States and Mexico and one from Guatemala, the latter with the southernmost distribution for the genus in the New World (Makarkin 1996, Monserrat 1998, Oswald 2018). Wesmaelius (Kimminsia) longipennis (Banks) is known from western (California) and south-western (Texas) United States (Klimaszewski and Kevan 1987a, Oswald 2018), as well as from the Mexican states of Chihuahua, Durango, and Morelos (Oswald et al. 2002), and is the only previously known species in Mexico. The New World species were treated more recently by Klimaszewski and Kevan (1987a, b), who revised the species from Alaska and Canada, and diagnosed the two subgenera proposed by Aspöck et al. (1980), *Kimminsia* Killington and *Wesmaelius* Krüger. This subdivision was questioned by Oswald (1993), on the grounds that *Kimminsia* may be paraphyletic, a position followed by Zhao et al. (2017), however there is no conclusive evidence yet to settle the validity of the subgenera. In this work, we describe a new species of *Wesmaelius* from Tlaxcala state, central Mexico, which belongs to the Transmexican Volcanic Belt biogeographic province (Morrone 2014). Following the classification scheme with subgenera (Aspöck et al. 1980; Klimaszewski and Kevan 1987a; Makarkin 1995, 1996), the new species agrees well with the subgenus Kimminsia, particularly because the elongate, narrowly rectangular male ectoproct, and the short female gonapophyses laterales. A key for the identification of the 15 currently known species of Wesmaelius (Kimminsia) from the New World is included; the only currently known species of Wesmaelius (W.) from the New World, the Nearctic Wesmaelius (W.) longifrons (Walker), may be identified with Klimaszewski and Kevan (1987b).

## Material and methods

The last four abdominal segments of both sexes were separated from the thorax and cleared in 10% potassium hydroxide (KOH) for 10 minutes at 60 °C. Structures were rinsed in distilled water with the aid of a 1 ml insulin syringe, placed in a glass microvial with glycerine, and then included with the specimen within a larger vial containing 96% ethanol. The terminology for wing and genitalia morphology follows Klimaszewski and Kevan (1987a). Observations were made under a Carl Zeiss Discovery V8 stereomicroscope and photographs were taken under a Carl Zeiss AxioZoom V16 stereomicroscope and a Leica Z16APO-A stereomicroscope with a camera Leica DFC490, both with automontage system. The collecting site, Municipality of Nanacamilpa, Tlaxcala State, has a temperate mesic climate with summer rains, with a mean annual temperature of 14.8 °C, and mean annual precipitation of 1729 mm (INEGI 2014). The holotype and one female paratype, both dissected, are deposited at the Colección Nacional de Insectos (CNIN) of the Instituto de Biología, UNAM, Mexico City; one male (undissected) and one female (dissected) paratype will be deposited at the National Museum of Natural History, Smithsonian Institution (NMNH), Washington, D.C. The Neuropterida species of the World platform (Oswald 2018) was helpful to track down all known *Wesmaelius* species, whose descriptions were compared in order to rule out synonymy. The key was constructed based on Klimaszewski and Kevan (1987a) and descriptions of other Wesmaelius (Kimminsia) species in the New World: Wesmaelius (K.) longipennis (Banks) from Mexico (Banks 1920, Carpenter 1940), and Wesmaelius (K.) magnus (Kimmins) from Guatemala (Kimmins 1928, Monserrat 1998). In the key, “fig.” refers to figures from literature, while “Fig.” refers to original figures.

## Taxonomy

### Wesmaelius (Kimminsia) nanacamilpa

Taxon classificationAnimaliaNeuropteraHemerobiidae

Marquez & Contreras sp. n.

http://zoobank.org/A877B409-F953-42B2-8BCE-901FB464FB84

Figs 1 , 2

#### Material examined.

Holotype: Male; MEXICO: Tlaxcala, Nanacamilpa de Mariano Arista, Comunidad San Felipe Hidalgo, Bosque Mágico de Piedra Canteada, Santuario de la Luciérnaga, 19°27'22.3"N, 98°36'02.0"W, 2839 m, 03.vi.2016, Marquez, Contreras, Ramírez, Mayorga, Luna, luz blanca, bosque de *Abies* [alcohol, genitalia dissected] (CNIN). Paratypes: same data as holotype, 1 female [alcohol, genitalia dissected] (CNIN), 1 female [alcohol, dissected] (NMNH); same data as holotype but 2855 m, 02.vi.2017, Marquez, Contreras, Ramírez, Luna, mercury vapor light, 1 male [alcohol, undissected] (NMNH).

#### Diagnosis.

**Figure 1.**
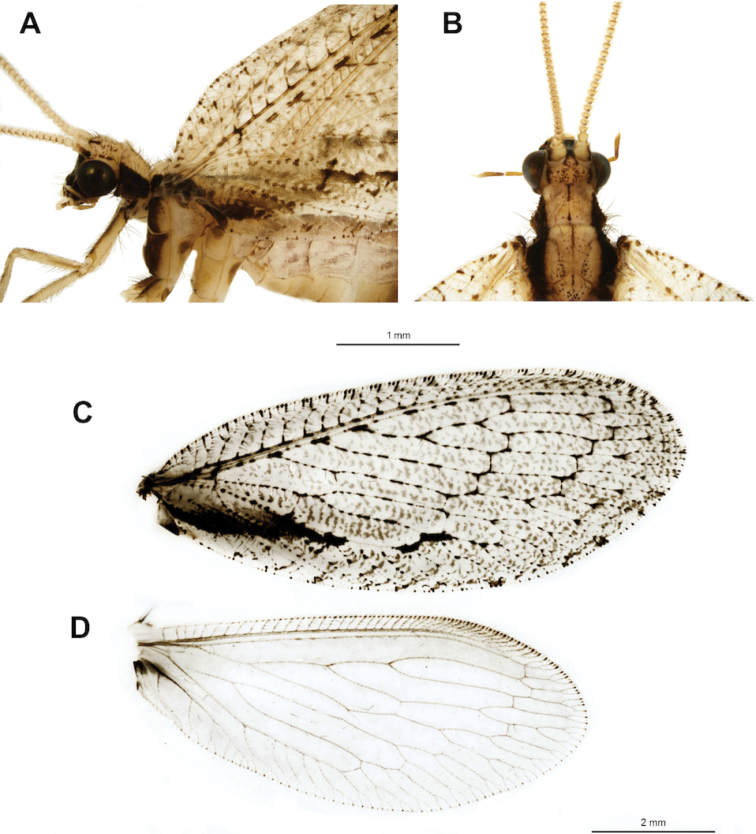
Wesmaelius (Kimminsia) nanacamilpa, sp. n., habitus and wings: **A** Head and thorax, lateral **B** Head and thorax, dorsal **C** Forewing **D** Hind wing. Scale bars: 1 mm (**A, B**), 2 mm (**C, D**).

**Figure 2.**
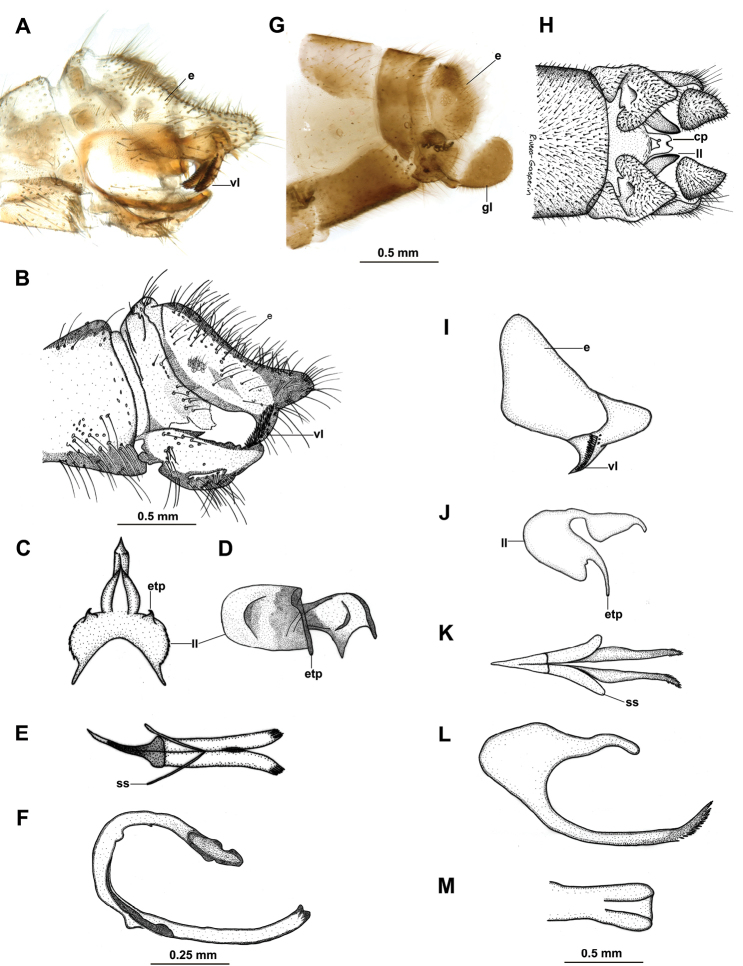
Wesmaelius (Kimminsia) nanacamilpa, sp. n., genitalia: **A, B** Male genitalia, lateral **C** Male gonarcus, dorsal **D** Male gonarcus, lateral **E** Male parameres, dorsal **F** Male parameres, lateral **G** Female genitalia, lateral **H** Female genitalia, ventral. Wesmaelius (Kimminsia) magnus (Kimmins): **I** Male ectoproct, lateral **J** male gonarcus, lateral **K** Male parameres, dorsal **L** Male parameres, lateral **M** Female subgenital plate, ventral. Abbreviations: cp, central plate; e, ectoproct; etp, entoprocessus; gl, gonapophyses laterales; ll, lateral lobe; ss, styliform sclerites; vl, ventral lobe. Scale bars: 0.5 mm (**A, B, G, H**), 0.25 mm (**C–F**), 0.5 mm (**I–M**); **I–M** redrawn from Monserrat (1998).

Head mostly dark brown (Fig. 1A), vertex yellowish with small brown spots (Fig. 1B); outer side of scape brown (Fig. 1A); colour pattern of pronotum a mid-longitudinal discontinuous line with adjacent small irregular spots (Fig. 1B); dark brown band running laterally along pronotum, mesothorax and extending towards the basal third of forewing (Fig. 1A, C). Male ectoproct with pecten strongly sclerotized (Fig. 2A, B); parameres diverging, distally with a narrow V-shape, styliform sclerites directed basally (Fig. 2E); gonarcus with short entoprocessus (Fig. 2C, D), lateral lobe broad (Fig. 2D); female lateral gonapophyses separate from ectoproct and ovoid (Fig. 2G), subgenital plate with large lateral lobe and central plate with broad basal incision (Fig. 2H), spermathecal duct moderately coiled (Fig. 2G).

#### Description.

*Dimensions*. Forewing length 9.2–9.5 mm, width 3.5–3.6 mm male (n = 2), length 9.6 mm, width 4.0 mm female (n = 2). *Body colour pattern*. Yellowish brown, with portions of head and thorax dark brown; wings patterned; abdomen yellowish, dark brown beyond fourth segment.

*Head*. Mostly dark brown. Vertex pale yellow with dark brown spots, two pale brown lines between antennae and two pale brown lines behind eyes (Fig. 1B); frons dark brow nearly black, clypeus reddish brown with transverse row of conspicuous setae on posterior margin (Fig. 1A); labrum reddish brown, gena and postorbital region dark brown, nearly black; male antennae 73–75-segmented (n = 2), female 73-segmented (n =1), scape yellowish, laterally brown, pedicel yellowish, flagellomeres yellowish with narrow brown ring at base (Fig. 1A); eyes black with metallic luster (Fig. 1A).

*Thorax*. Contrasting yellowish brown with dark brown. Pronotum yellowish with two discontinuous mid-longitudinal lines with adjacent small irregular spots (Fig. 1B); dark brown band running along pronotum, mesothorax and extending towards the basal third of forewing (Fig. 1A, C); mesonotum yellowish, small dark brown spots forming a pattern (Fig. 1B); frontal side of mesothorax blackish brown (Fig. 1A). Legs mostly yellowish brown, anterior side of forefemur and midcoxa brown, hind legs mostly yellowish.

*Wings*. Forewing narrowly oval; patterned with pale brown maculation; pterostigma pinkish, undefined; costal area narrow, wider at basal third; gradate series equally distanced; dark brown, nearly black irregular band along anal and cubital proximal third of wing; irregular elongate maculation at medial-cubital area in middle of wing (Fig. 1C). Hind wing broadly oval, mostly hyaline, without maculation except portions of costal and anal areas; patch of setae at base, before costal vein (Fig. 1D).

*Abdomen*. Mostly pale brown, dark brown beyond fourth segment, particularly dorsally.

*Male genitalia*. Ninth tergum narrow dorsally, broad basally, concave at upper posterior margin in lateral view (Fig. 2A, B); ectoproct subrectangular, with a prominent posterodorsal lobe, ventral lobe short with sclerotized teeth, 12 or 13 trichobothria (Fig. 2A, B). Gonarcus rounded, with mediuncus sharp, spine-like in dorsal view, short in lateral view (Fig. 2C, D); arcus with anterior projection subtriangular; lateral lobes convex in dorsal view (Fig. 2C), amply subquadrate in lateral view (Fig. 2D), proximal corners sharp (Fig. 2C); entoprocessus short, far from mediuncus (Fig. 2C, D). Parameres bar-like, diverging distally with a narrow V-shape, tips sclerotized with small teeth, base slender, strongly sclerotized, styliform sclerites directed basally (Fig. 2E, F).

*Female genitalia*. Gonapophyses laterales separate from ectoproct, ovoid, less sclerotized at proximal side; ectoproct short, subrectangular, bearing 12–14 trichobothria (Fig. 2G); subgenital plate with central plate incised apically, conspicuously incised at base, internal margins of basal incision serrate; lateral lobes narrowly lanceolate, longer than central plate (Fig. 2H); spermatheca with sclerotized duct moderately coiled (Fig. 2G).

#### Variation.

Females were slightly larger than males, also females had a stronger colour pattern than males.

#### Etymology.

Named after Nanacamilpa, the municipality of Tlaxcala State where the specimens were collected, meaning ‘field of mushrooms’ in Nahuatl.

#### Ecology.

Specimens were attracted to white light (domestic light bulb) and mercury vapour light in an *Abiesreligiosa* (Sacred Fir) forest, during the rainy season (early June). Specimens of *Wesmaelius* are generally captured in conifer forests, maintaining their activity at low temperatures with small population sizes (Klimaszewski and Kevan 1987b; Monserrat 1998, 2015).

## Discussion

The new species is similar to Wesmaelius (Kimminsia) magnus (Kimmins) in wing colour pattern, including a pinkish pterostigma and a dark spot at the MP-CuA inner gradates, however W. (K.) magnus lacks the conspicuous dark band at the base of forewing (Kimmins 1928, Monserrat 1998); both have the head vertex with dark brown punctuations, less dense in W. (K.) magnus (Monserrat 1998). Wesmaelius (K.). *longipennis* (Banks) appears to have a similar colour pattern, but the face has numerous red specks (Carpenter 1940). Regarding external genitalia, the new species is similar to W. (K.) longipennis, W. (K.) magnus, W. (K.) involutus (Carpenter), W. (K.) brunneus (Banks), W. (K.) yuconensis Klimaszewski and Kevan, and W. (K.) pretiosus (Banks) in the shape of the ectoproct, however considering the morphology of the parameres and gonarcus, the new species is distinct from the other known species. At present, it would be difficult to propose a possible sister species. In general, the anterior projection of the gonarcus is similar in dorsal view between W. (K.) magnus from Guatemala and the new species, yet in detail there are marked differences. The gonarcus is broader in the new species (Fig. 2C, D), entoprocessus is shorter (Fig. C, D), and the parameres are bar-like, with uniform width in the new species (Fig. 2E, F), while slightly curvy and widened at the base in W. (K.) magnus (Fig. 2K, L). Females of the new species might be characteristic based on the shape of the subgenital plate, with a marked basal incision with an internal irregular margin (Fig. 2H), however not clearly comparable with W. (K.) magnus at present (Fig. 2M); the sclerotized spermathecal duct is less coiled and shorter in the new species (Fig. 2G), as compared with W. (K.) magnus (Monserrat 1998, fig. 17). A study focused on the brown lacewings of cold mountainous habitats in Mexico, may increase the number of recorded *Wesmaelius* species in the country.

### Key to New World species of Wesmaelius (Kimminsia) Killington (males)

**Table:** 

1	First fork of anterior cubitus (CuA) at or extremely close to mediocubital inner gradate cross-vein (m+cuig) (Klimaszewski and Kevan 1987a: figs 157A, 158A, 165A)	**2**
–	First fork of anterior cubitus (CuA) distal to mediocubital inner gradate cross-vein (m+cuig) (Klimaszewski and Kevan 1987a: figs 159A, 160A-163A, 164A, 166A, 167A)	**6**
2	Forewing apparently immaculate or little maculate, veins unicolorous without variegation (Klimaszewski and Kevan 1987a: fig. 165A)	***W.brunneus* (Banks)**
–	Forewing with distinct maculation pattern, veins variegated pale and dark (Klimaszewski and Kevan 1987a: figs 157A, 158A)	**3**
3	Forewing with apex and ventral (posterior) margin broadly rounded, costal area gradually narrowed at base (Fig. 1C)	**4**
–	Forewing with apex and ventral (posterior) margin slightly rounded, costal area abruptly narrowed at base (Klimaszewski and Kevan 1987a: figs 157A, 158A)	**5**
4	Vertex with scarce punctuation (Monserrat 1998: fig. 11); parameres deeply divided, with apex amply denticulate, styliform sclerites directed apically (Fig. 2K, L)	***W.magnus* (Kimmins)**
–	Vertex with dense punctuation (Fig. 1B); parameres divided distally, with apex narrowly denticulate, styliform sclerites directed basally (Fig. 2E, F)	***W.nanacamilpa* Marquez & Contreras, sp. n.**
5	Ectoproct with large ventral lobes, gonarcus with arcus and mediuncus close in lateral and dorsal views, parameres with styliform sclerites large-convex (Klimaszewski and Kevan 1987a: figs 64–68)	***W.coloradensis* (Banks)**
–	Ectoproct with short ventral lobes, gonarcus with arcus and mediuncus distal in lateral and dorsal views, parameres with styliform sclerites small, triangular (Klimaszewski and Kevan 1987a: figs 24, 25, 27–30)	***W.involutus* (Carpenter)**
6	Ectoproct with long ventral lobe (Klimaszewski and Kevan 1987a: figs 45, 46, 74, 75)	**7**
–	Ectoproct with short ventral lobe (Klimaszewski and Kevan 1987a: figs 8, 9, 56, 57)	**10**
7	Ectoproct with ventral lobe twisted, directed first inwards and then caudad, exceptionally long (Klimaszewski and Kevan 1987a: figs 45, 74)	**8**
–	Ectoproct with ventral lobe curved, long, directed only once inwards (Klimaszewski and Kevan 1987a: figs 8, 56)	**9**
8	Ectoproct strongly narrowing apically, with twisted ventral lobe (Klimaszewski and Kevan 1987a: figs 74, 75)	***W.furcatus* (Banks)**
–	Ectoproct elongate, subquadrate, with ventral lobe strongly curved inwards (Klimaszewski and Kevan 1987a: figs 45, 46)	***W.subnebulosus* (Banks)**
9	Ectoproct with ventral lobe elongate, subquadrate, gonarcus with mediuncus and entoprocessus close, compact and broad (Klimaszewski and Kevan 1987a: figs 56, 57, 60)	***W.posticatus* (Banks)**
–	Ectoproct with ventral lobe elongate-narrow, gonarcus with mediuncus and entoprocessus distal and extended, separate and slim (Klimaszewski and Kevan 1987a: figs 8, 14)	***W.nervosus* (Fabricius)**
10	Body and wings marked with red specks; gonarcus with elongate arcus (Carpenter 1940: fig. 20C)	***W.longippenis* (Banks)**
–	Body and wings differently marked, without red specks; gonarcus with short arcus (Klimaszewski and Kevan 1987a: fig. 11)	**11**
11	Gonarcus with two lateral emarginations in lateral view (Klimaszewski and Kevan 1987a: fig. 111)	***W.constrictus* (Parfin)**
–	Gonarcus without lateral emarginations (Klimaszewski and Kevan 1987a: fig. 104)	**12**
12	Parameres continuous (Klimaszewski and Kevan 1987a: fig. 101)	***W.yuconensis* (Klimaszewski & Kevan)**
–	Parameres apparently subdivided (Klimaszewski and Kevan 1987a: fig. 118)	**13**
13	Arcus curved in lateral view, triangular in dorsal view (Klimaszewski and Kevan 1987a: figs 103, 104)	***W.fumatus* (Carpenter)**
–	Arcus straight in lateral view, subtriangular in dorsal view (Klimaszewski and Kevan 1987a: figs 107, 116)	**14**
14	Frons dark brown to almost black. Arcus with small tooth at base in lateral view (Klimaszewski and Kevan 1987a: fig. 107)	***W.schwarzi* (Banks)**
–	Frons yellow to yellowish-brown. Arcus with protuberance at base in lateral view (Klimaszewski and Kevan 1987a: fig. 116)	***W.pretiosus* (Banks)**

## Supplementary Material

XML Treatment for Wesmaelius (Kimminsia) nanacamilpa
